# Breastfeeding and weaning practices among Hong Kong mothers: a prospective study

**DOI:** 10.1186/1471-2393-10-27

**Published:** 2010-05-29

**Authors:** Marie Tarrant, Daniel YT Fong, Kendra M Wu, Irene LY Lee, Emmy MY Wong, Alice Sham, Christine Lam, Joan E Dodgson

**Affiliations:** 1 School of Nursing, Li Ka Shing Faculty of Medicine, 21 Sassoon Road, Pokfulam, Hong Kong; 2 School of Public Health, Li Ka Shing Faculty of Medicine, 21 Sassoon Road, Pokfulam, Hong Kong; 3 Dept. of Obstetrics and Gynaecology, Queen Mary Hospital, Pokfulam, Hong Kong; 4 Dept. of Health and Physical Education, The Hong Kong Institute of Education, Tai Po, New Territories, Hong Kong; 5 Kwong Wah Hospital, Kowloon, Hong Kong; 6 Dept. of Obstetrics and Gynaecology, Queen Elizabeth Hospital, Kowloon, Hong Kong; 7 College of Nursing and Healthcare Innovation, Arizona State University, 500 N. 3rd Street, Phoenix, AZ, USA

## Abstract

**Background:**

Breastfeeding provides optimal and complete nutrition for newborn babies. Although new mothers in Hong Kong are increasingly choosing to breastfeed their babies, rates of exclusive breastfeeding are low and duration remains short. The purpose of this study was to describe the breastfeeding and weaning practices of Hong Kong mothers over the infant's first year of life to determine the factors associated with early cessation.

**Methods:**

A cohort of 1417 mother-infant pairs was recruited from the obstetric units of four public hospitals in Hong Kong in the immediate post-partum period and followed prospectively for 12 months or until weaned. We used descriptive statistics to describe breastfeeding and weaning practices and multiple logistic regression to investigate the relationship between maternal characteristics and breastfeeding cessation.

**Results:**

At 1 month, 3 months, 6 months and 12 months only 63%, 37.3%, 26.9%, and 12.5% of the infants respectively, were still receiving any breast milk; approximately one-half of breastfeeding mothers were exclusively breastfeeding. Younger mothers, those with a longer duration of residence in Hong Kong, and those returning to work postpartum were more likely to wean before 1 month. Mothers with higher education, previous breastfeeding experience, who were breastfed themselves and those who were planning to exclusively breastfeed and whose husbands preferred breastfeeding were more likely to continue breastfeeding beyond 1 month. The introduction of infant formula before 1 month and returning to work postpartum were predictive of weaning before 3 months.

**Conclusions:**

Breastfeeding promotion programs have been successful in achieving high rates of breastfeeding initiation but the focus must now shift to helping new mothers exclusively breastfeed and sustain breastfeeding for longer.

## Background

Breastfeeding provides optimal and complete nutrition for newborn babies. The benefits of breastfeeding to both the infant and the mother have been widely recognized [1] and the health risks associated with infant formula feeding are increasingly documented [2-4]. During infancy, breastfeeding protects against infectious disease [5-7] and long term, breastfeeding is associated with benefits in several areas, such as cardiovascular risk factors [8], intellectual capacity [9,10], and allergy [11,12]. Even in the developed world, breastfeeding offers substantial health benefits to infants and young children [6,13,14]. Both the World Health Organization (WHO) [1] and the American Academy of Pediatrics [5] have recommended exclusive breastfeeding for the first six months of life, with continued breastfeeding up to 12 months of age or longer along with the introduction of solid foods.

Women in Hong Kong, like women in many other industrialized countries, are increasingly choosing to breastfeed their infants. Although more than 70% of all new mothers now initiate breastfeeding [15], up from 19% in 1981 [16] and 37% in 1997 [17], Hong Kong still has low breastfeeding rates when compared with other developed countries [18,19]. Despite the recommendation of the Hong Kong Department of Health, which like the WHO [1] recommends exclusive breastfeeding until 6 months of age [20], and active promotion of breastfeeding by the Hong Kong Government and various other organizations [15], both the duration of exclusive breastfeeding and the total duration of breastfeeding remain below the WHO recommendations [1,21]. Few Hong Kong women exclusively breastfeed and most stop breastfeeding within the first few months postpartum [22]. A population-based birth cohort study done in 1997 found that only 20.4% and 10.3% of infants were still breastfeeding at 1 month and 3 months of age, respectively [17]. Another large breastfeeding survey showed that from 1997 to 2000, 35-39% of women breastfed for at least 1 month, 22-28% breastfed for 4 months, and 15-19% breastfed for at least 6 months [16]. A longitudinal non-population based study found that about 20% of women continued to breastfeed for 6 months or longer [22].

The high breastfeeding attrition rate in Hong Kong has been attributed to sociocultural variables such as lack of family, community, and workplace support for breastfeeding [22,23]. As in many Western societies, breastfeeding in public is often perceived as embarrassing and cultural values have made formula feeding the norm. While there have been an increasing number of studies on breastfeeding among Hong Kong mothers over the past decade, few studies have described breastfeeding and weaning practices in Hong Kong in any detail. Most larger-scale studies that have been done in Hong Kong are now outdated as they were done when breastfeeding rates were substantially lower than today [17,24,25]. Furthermore, while exclusive breastfeeding rates are known to be low and breastfeeding duration remains short, little research has documented breastfeeding practices over the first year of life and the self-identified reasons for weaning. This longitudinal prospective cohort study of mother-infant pairs provides more up-to-date information on the breastfeeding and weaning practices in Hong Kong and describes the differences in these patterns from those of other developed countries. In addition, this study highlights some possible explanations for the low prevalence of continued breastfeeding in the population, which have significant public health implications.

### Study Aims

The aim of this study was to describe the breastfeeding and weaning practices of Hong Kong mothers over the infant's first year of life and to determine the factors associated with early cessation. The specific objectives were to:

1. Describe the duration of exclusive breastfeeding and any breastfeeding

2. Describe patterns of exclusive and partial breastfeeding

3. Examine the relationship between continued breastfeeding and key sociodemographic characteristics

4. Identify the predominant reasons for weaning at different time periods during the infant's first year.

## Methods

### Participants

This study used a prospective longitudinal cohort design. New mothers admitted to the obstetric units of four geographically distributed public hospitals in Hong Kong were recruited into the study in the immediate post-partum period. Hong Kong has 8 public and 9 private hospitals that deliver obstetric care. In 2008, public hospital births accounted for 52.7% of all births in Hong Kong [15]. From June 2006 to June 2007, we recruited 1417 mother-infant pairs from the four study sites. New mothers were eligible to participate in the study if they met the following selection criteria: (1) intention to breastfeed; (2) singleton pregnancies; (3) Cantonese speaking; (4) Hong Kong residents; and (5) no serious medical or obstetrical complications. Mothers who were not planning to breastfeed (i.e., those who were planning to exclusively formula feed) were not recruited into the study. In addition, participants were excluded from the study if their baby: (1) was born before 37 weeks gestation, (2) had an Apgar score of less than eight at five minutes, (3) had a birth weight of less than 2500 gms, (4) was born with any severe medical conditions or congenital malformations, (5) was placed in the special care nursery for more than 48 hours after delivery, or (6) was placed in the neonatal intensive care unit after delivery.

### Data Sources

#### Baseline

Breastfeeding mothers were recruited during their post-partum stay and were asked to complete a basic sociodemographic questionnaire. The questionnaire included information about breastfeeding intentions (i.e., duration and exclusivity), prenatal education, previous breastfeeding experience, family breastfeeding support, family composition, and basic socioeconomic indicators (i.e., income, education, and occupation). The demographic questionnaire was translated into Chinese (Cantonese) by an expert translator. To ensure the accuracy of the translation, back-translation of the Chinese version of the instrument into English was performed by a different translator following established procedures [26,27]. The questionnaire used standardized categories for assessing basic demographic characteristics [28]. Any discrepancies between the original English version and the back translated English version were examined and modifications were made so that the questions were translated accurately.

Other in-hospital data collected included maternal and infant health data (i.e., previous obstetric history, delivery data, and basic infant health data) and infant feeding data. These data were abstracted by the study research nurse from the participant's medical record and the 24-hour intake and output record during the hospital stay.

#### Follow-up

Follow-up infant feeding and weaning data were collected after hospital discharge through telephone interviews at 1, 2, 3, 6, 9, and 12 months post-partum or until weaned, whichever came first. During the follow-up interviews, milk-feeding patterns were assessed by asking participants if their child was still currently receiving any breast milk, and if so, to classify the current method of milk feeding as either breast milk only or mixed breastmilk and formula, with clear explanations of each provided. Participants who were no longer breastfeeding were classified as full formula feeding. Additionally, breastfeeding participants were asked to recall for the past 24 hours how many breast milk feedings were given at the breast, how many expressed breast milk feedings were given and how many artificial milk feeds were given. Beginning at the 6-month follow-up we asked the mother specifically about the milk-only portion of the infant's diet, not solid foods. Therefore, giving breast milk only at 6 months and beyond could include complementary food but not artificial formula or other milk replacements. Weaning data was collected from the mother at the follow-up interview upon the discontinuation of all breastfeeding and included the total duration of both any breastfeeding and exclusive breastfeeding and the reasons for weaning. Participants were asked an open-ended question to identify the reasons for breastfeeding cessation. The participant could state any number of reasons for weaning and no list of reasons was provided. After identifying the reasons for weaning, the participant was further asked which of the provided reasons was the primary reason for weaning. No further data was collected after the mother had weaned.

### Key Measures

Breastfeeding status was defined as exclusive or non-exclusive. Infants were considered exclusively breastfed if they received no breast milk substitutes (other than vitamins or medications) and were considered non-exclusively breastfed if they were supplemented with infant-formula and/or other breast milk substitutes [29]. The feeding of other non-milk weaning liquids to young infants, such as teas and water, is no longer common in Hong Kong [30] and therefore we did not assess the prevalence of predominant breastfeeding. For non-exclusively breastfeeding infants, we computed the proportion of total daily feeds which were breast milk. Infants were then classified as exclusive breastfeeding, more than two-thirds breast milk, one third to two thirds breast milk, less than one third breast milk, or no breast milk [31]. Once it was ascertained that the child was completely weaned off breast milk, it was assumed thereafter that the child was receiving no breast milk.

### Statistical Analysis

Descriptive statistics were used to describe the duration and patterns of breastfeeding. We grouped mothers according to the age when they stopped breastfeeding: <1, 1 to <3, 3 to <6, 6 to <9, 9 to <12, and ≥12 months. We used chi-square tests and one-way analysis of variance to assess the relationship between the infant's weaning age and various maternal sociodemographic characteristics and we used chi-square tests to assess the relationship between the infant's weaning age and the reasons for weaning. We used multivariable Logistic Regression to give the adjusted Odds Ratios (aORs) of weaning at two time intervals (<1 month and <3 months) as these two periods accounted for almost 2/3 of all participants. The Hosmer-Lemeshow test [32] was used to assess the adequacy of the analyses and variance inflation factor (VIF) was used to assess for multicollinearity [33]. All analyses were conducted using Stata version 9.2 statistical software (Stata Corp, College Station, TX) [34]. Ethical approval for the study was obtained from the Institutional Review Board of The Li Ka-Shing Faculty of Medicine, University of Hong Kong and the participating study sites and informed written consent was obtained from all participants.

## Results

There were 1417 mother-infant pairs from the four study sites in the baseline sample (Site A = 419; Site B = 378; Site C = 302; Site D = 318). After recruitment we excluded eight participants because they subsequently did not meet the study eligibility criteria (i.e., the baby was transferred to the neonatal intensive care unit), five participants with substantial missing data, and 87 (6.1%) participants with whom we had no further contact after hospitalisation. When compared with those who stayed in the study, participants with whom we had no contact after hospitalisation were more likely to have lower monthly family income (χ^2 ^= 10.44, p < .01) and to have lived in Hong Kong for <5 years (χ^2 ^= 20.17, p < .001). There were no differences in maternal age (χ^2 ^= 3.16, p = .37) or education (χ^2 ^= 2.74, p = .25).

**Figure 1:**
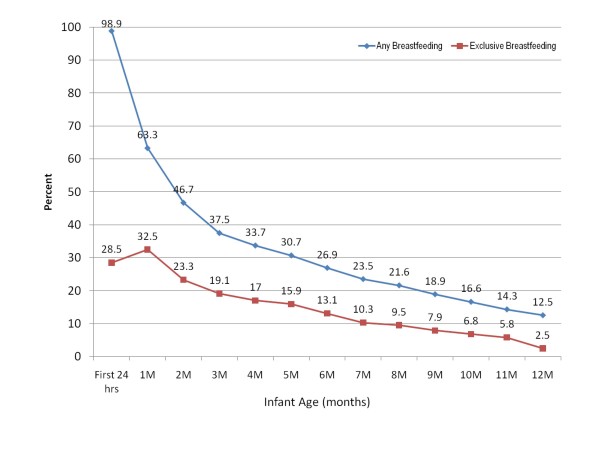
**The proportion of mothers still providing any breastfeeding and exclusively breastfeeding at monthly time intervals**.

**Figure 2:**
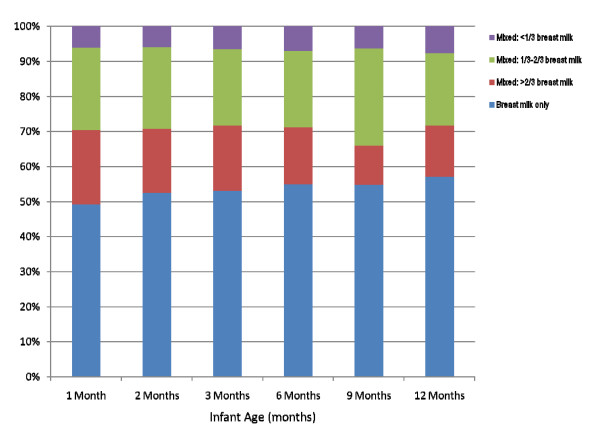
**The proportion of milk and formula feedings among breastfeeding mothers across the first year of life**. Data refers only to the milk-based infant diet and therefore after 6 months of age would also include the feeding of complementary foods along with breastmilk and infant formula. Legend: Sample sizes were as follows: 1 month, n = 834; 2 months, n = 615; 3 months, n = 494, 6 months, n = 354; 9 months, n = 249; 12 months, n = 165.

**Figure 3:**
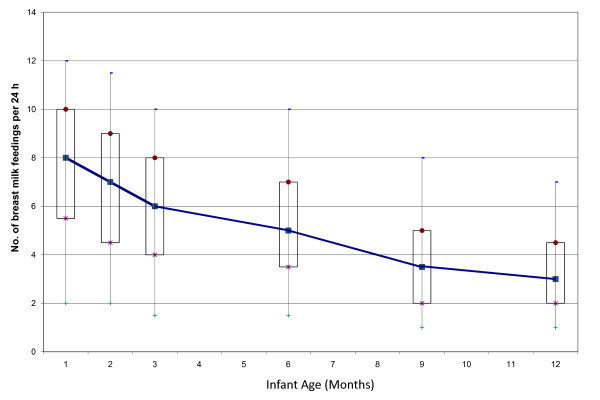
**Median, interquartile, and 95th percentile ranges of frequency of breast milk feedings per 24 hours according to infant age**. Sample sizes were as follows: 1 month, n = 764; 2 months, n = 516; 3 months, n = 460, 6 months, n = 316; 9 months, n = 227; 12 months, n = 140.

**Figure 4:**
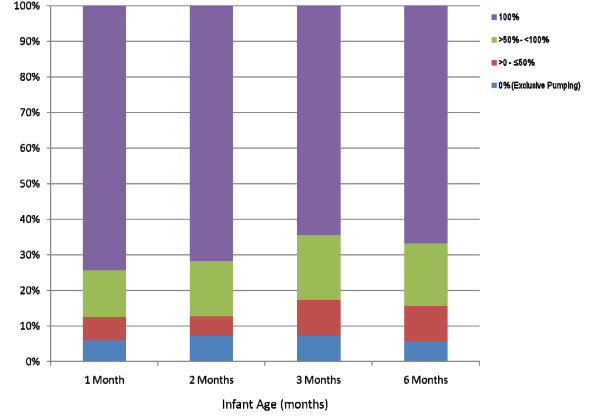
**The proportion of breast milk feedings given directly at the breast over the first 6 months of life**. Sample sizes were as follows: 1 month, n = 791; 2 months, n = 561; 3 months, n = 467, 6 months, n = 330.

The final analysis included 1317 (92.9%) mother-infant pairs. Of these 1317 participants, 1119 (85.0%) stopped breastfeeding before the end of the study at 12 months, 165 (12.5%) were still breastfeeding at 12 months, and 33 (2.5%) were still breastfeeding when they were lost to follow-up or dropped out of the study. These 33 participants were included in all analyses, unless otherwise specified, and were assumed to have weaned at the time of last contact. At 1 month, 3 months, 6 months and 12 months only 63%, 37.3%, 26.9%, and 12.5% of the infants respectively, were still receiving any breast milk (Figure 1). At most time points, approximately one-half of breastfeeding mothers were giving only breast milk to their infants. Figure 2 shows the proportion of breast milk and infant formula for those infants still breastfeeding. Over the first year, the proportion of infants' milk-based diet that consisted of only breast milk increased marginally from 49.0% at one month to 54.8% at 12 months. Only a small proportion of infants (<8%), however, received token amounts of breast milk (<1/3 breast milk) at any time point. The median number of breast milk feedings dropped from 8 per day in the first month to 6 per day in the third month and to 3 per day by 12 months (Figure 3). Among mothers who were still breastfeeding at one month, 5.9% were pumping exclusively, meaning that the infants were never fed directly from the breast, and 74.5% were fed 100% directly from the breast (Figure 4). The proportion of women expressing any breast milk ranged from 25.5 to 35.3% across the first six months with a slight increase after two months.

**Figure 5:**
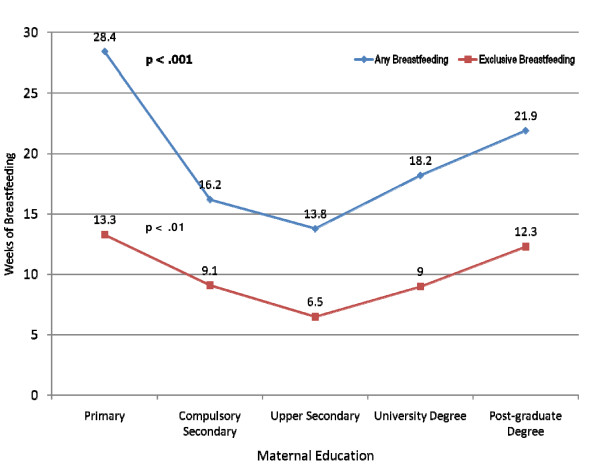
**Weeks of any and exclusive breastfeeding by level of maternal education**. Compulsory Secondary is Form 3 and Upper Secondary is Form 7. Participants lost-to-follow-up (n = 33) were excluded. The sample size for this analysis was 1284.

**Figure 6:**
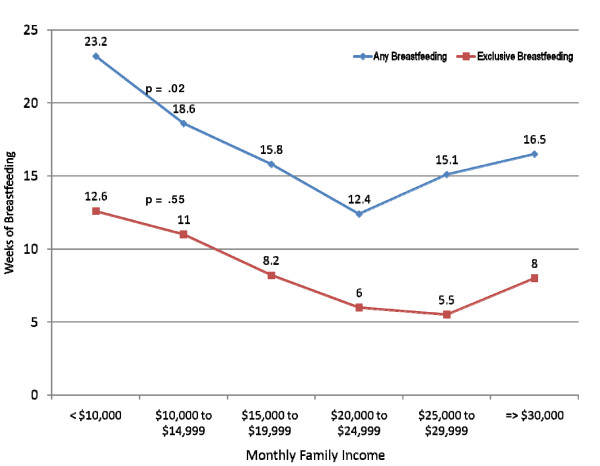
**Weeks of any and exclusive breastfeeding by level of family income**. Values are in Hong Kong Dollars (1 USD = 7.78 HKD). Participants lost-to-follow-up (n = 33) and those with missing income data (n = 35) were excluded. The sample size for this analysis was 1249.

The characteristics of the study sample according to the infant's age at weaning are presented in Table 1. Demographic characteristics associated with earlier weaning include younger maternal age, being a first-time mother, living in Hong Kong for ≥5 years, the mother not having been breastfed herself, the husband's preference for infant formula or mixed feeding, having an emergency caesarean section, and returning to work post-partum. Maternal education and family income showed a more complex interaction with weaning time. Participants in both the middle education and family income levels had earlier weaning than participants with low and high levels of both education and family income (Figures 5 and 6). Mothers with only primary level education has a mean breastfeeding duration of 28.4 weeks compared with 13.8 weeks for those with secondary education and 21.9 weeks for those with a post-graduate degree (p < .001 for quadratic effects) (Figure 5). Additionally, mothers in the lowest income group breastfed for a mean duration of 23.2 weeks compared with only 12.4 weeks for those in the fourth income group and 16.5 weeks for those in the highest income group (p = .02 for quadratic effects) (Figure 6).

**Table 1:** Characteristics of the study sample according to infants' age at weaning

		Infants' age in months when breastfeeding was completely stopped					
		
Characteristic	Total (*N *= 1317) %	<1 (*n *= 483) %	1 to <3 (*n *= 340) %	3 to <6 (*n *= 140) %	6 to <9 (*n *= 105) %	9 to <12 (*n *= 84) %	≥12 (*n *= 165) %
Age,‡ y							
18-24	6.6	9.9	5.6	2.9	4.8	3.6	4.9
25-29	22.9	24.8	23.5	19.3	21.0	22.6	20.6
30-34	46.2	45.6	44.7	50.7	50.5	46.4	44.9
≥35	24.2	19.7	26.2	27.1	23.8	27.4	29.7
Parity†							
Primiparous	59.8	70.6	62.1	52.9	54.3	45.2	40.6
Multiparous	40.2	29.4	37.9	47.1	45.7	54.7	59.4
Maternal education†							
Primary	3.6	2.7	1.8	1.4	4.8	7.1	9.1
Compulsory secondary	20.3	20.9	19.7	20.0	21.9	23.8	17.0
Upper secondary	37.4	48.2	33.8	26.4	30.5	28.6	31.5
University degree	35.5	25.5	41.7	49.3	41.0	35.7	36.4
Post-graduate degree	3.3	2.7	2.9	2.9	1.9	4.8	6.1
Monthly Family income (HKD)†^ab^							
< $10,000	8.7	6.8	6.4	11.3	6.9	12.2	16.1
$10,000-14,999	13.7	12.9	12.5	12.8	18.8	18.3	13.6
$15,000-$19,999	10.9	12.9	8.8	7.5	11.9	18.3	8.0
$20,000-$24,999	11.6	15.0	12.2	6.0	8.9	8.5	8.6
$25,000-$29,999	10.9	12.5	10.9	7.5	12.9	3.7	11.7
=> $30,000	44.2	40.1	49.2	54.9	40.6	39.0	42.0
Length of residence in Hong Kong†							
< 5 years	11.2	4.8	10.0	14.3	19.1	23.8	18.2
≥ 5 years	88.8	95.2	90.0	85.7	81.0	76.2	81.8
Mother planning to exclusively breastfeed†							
No	35.9	48.0	38.2	25.7	27.6	26.2	14.6
Yes	64.1	52.0	61.8	74.3	72.4	73.8	85.5
Was mother breastfed†							
No	54.1	62.5	54.7	47.1	44.8	46.4	44.2
Yes	45.9	37.5	45.3	52.9	55.2	53.6	55.8
Previous breastfeeding experience†							
No	66.4	79.1	67.9	58.6	59.1	50.0	46.1
Yes	33.6	20.1	32.1	41.4	41.0	50.0	53.9
Husband feeding preference†							
Breastfeeding	61.7	50.7	60.3	70.7	75.2	71.4	75.8
No preference	19.0	22.2	17.1	19.3	16.2	15.5	17.0
Infant formula or mixed	19.3	27.1	22.7	10.0	8.6	13.1	7.3
Emergency caesarean section‡							
No	88.7	86.3	87.7	89.3	90.5	97.6	91.5
Yes	11.3	13.7	12.4	10.7	9.5	2.4	8.5
Return to work post-partum†							
No	26.3	18.4	21.8	25.0	36.2	47.6	42.4
Yes	73.7	81.6	78.2	75.0	63.8	52.4	57.6

‡ *p *< .05

† *p *< .001

^a ^1 USD = 7.78 HKD

^c ^36 participants had missing family income data

Table 2 presents the results of the multivariable logistic regression examining the aORs between sociodemographic characteristics and early weaning. In the multivariable analysis predictors of weaning before 1 month were younger maternal age (aOR = 1.84; 95% CI 1.05 to 3.21), longer duration of residence in Hong Kong (aOR = 2.56; 95% CI 1.50 to 4.38), and returning to work post-partum (aOR = 1.75; 95% CI 1.25 to 2.46). Factors associated with continuation of breastfeeding included a university degree (aOR = 0.36; 95% CI 0.27 to 0.50) or a post-graduate degree (aOR = 0.44; 95% CI 0.21 to 0.90), planning to exclusively breastfeed (aOR = 0.66; 95% CI 0.50 to 0.86), previous breastfeeding experience (aOR = 0.36; 95% CI 0.22 to 0.61), mothers who were breastfed themselves (aOR = 0.74; 95% CI 0.57 to 0.95) and those whose husbands preferred breastfeeding (aOR = 0.71; 95% CI 0.51 to 0.98). Among those who were still breastfeeding after the first month, factors negatively associated with continued breastfeeding beyond three months included the husband's preference for infant formula (aOR = 2.39; 95% CI 1.31 to 4.34) and returning to work (aOR = 2.06; 95% CI 1.33 to 3.18). Additionally, supplementation with infant formula at one month had a clear dose-response effect on breastfeeding with the aORs of weaning increasing as the proportion of breastfeeding decreased from high-partial breastfeeding (aOR = 2.00; 95% CI 1.31 to 3.05) to low-partial breastfeeding (aOR = 13.8; 95% CI 5.70 to 33.63). The results of the Hosmer-Lemeshow goodness of fit tests for the two logistic regression models were .30 and .23, respectively, indicating that the models are a good fit for the data. VIF values indicate a low degree of multicollinearity.

**Table 2:** Adjusted^a ^odds ratios of weaning at different time intervals according to maternal demographic variables

	Breastfed for <1 month (*n *= 1281)^c^		Breastfed for <3 months (*n *= 765)^d^	
	
Characteristic	aOR^a^	95% CI	aOR^a^	95% CI
Age, y				
18-24	1.84	1.05 to 3.21	1.56	0.66 to 3.66
25-29	1.20	0.82 to 1.75	1.34	0.82 to 2.19
30-34	1.17	0.85 to 1.62	0.86	0.57 to 1.30
≥35	1	--	1	--
Parity				
Primiparous	1.52	0.92 to 2.51	1.11	0.53 to 2.32
Multiparous	1	--	1	--
Maternal Education				
Primary	0.91	0.41 to 2.02	0.55	0.19 to 1.64
Compulsory secondary	0.76	0.54 to 1.08	1.05	0.64 to 1.73
Upper secondary	1	--	1	--
University degree	0.36	0.27 to 0.50	0.82	0.53 to 1.27
Post-graduate degree	0.44	0.21 to 0.90	0.69	0.26 to 1.80
Monthly Family income (HKD)^b^				
< $10,000	0.79	0.42 to 1.50	0.55	0.23 to 1.30
$10,000-14,999	0.77	0.46 to 1.29	0.68	0.33 to 1.39
$15,000-$19,999	0.89	0.53 to 1.47	0.63	0.29 to 1.34
$20,000-$24,999	1	--	1	--
$25,000-$29,999	0.76	0.46 to 1.26	0.73	0.35 to 1.52
=> $30,000	0.82	0.54 to 1.23	0.77	0.43 to 1.40
Length of residence in Hong Kong				
< 5 years	1	--	1	--
≥ 5 years	2.56	1.50 to 4.38	0.83	0.46 to 1.48
Mother planning to exclusively breastfeed				
No	1	--	1	--
Yes	0.66	0.50 to 0.86	0.94	0.63 to 1.41
Was mother breastfed				
No	1	--	1	--
Yes	0.74	0.57 to 0.95	0.84	0.59 to 1.20
Previous breastfeeding experience				
No	1	--	1	--
Yes	0.36	0.22 to 0.61	0.90	0.43 to 1.87
Husband feeding preference				
Breastfeeding	0.71	0.51 to 0.98	1.04	0.66 to 1.64
No preference	1	--	1	--
Infant formula or mixed	1.28	0.87 to 1.89	2.39	1.31 to 4.34
Emergency caesarean section				
No	1	--	1	--
Yes	1.37	0.93 to 2.02	1.42	0.81 to 2.51
Return to work post-partum				
No	1	--	1	--
Yes	1.75	1.25 to 2.46	2.06	1.33 to 3.18
% of breastfeeding at 1 month				
Exclusive breastfeeding	--	--	1	--
High partial breastfeeding	--	--	2.00	1.31 to 3.05
Medium partial breastfeeding	--	--	5.25	3.36 to 8.20
Low partial breastfeeding	--	--	13.8	5.70 to 33.63
% of breastfeeds given at breast				
100%	--	--	1	--
50 to <100%	--	--	0.78	0.47 to 1.30
<50%	--	--	1.54	0.90 to 2.63

^a ^Adjusted for all variables shown

^b ^1 USD = 7.78 HKD

^c ^36 participants were excluded from the multivariable analyses because of missing family income data

^d ^69 participants were excluded from the multivariable analyses because of missing data

The primary reasons for weaning at the selected time intervals are shown in Table 3. The most common reason for weaning, "insufficient milk," was identified almost equally across the first year. The second most common reason for weaning, "returning to work," was identified much more frequently by women who weaned from 1 to <3 months or from 3 to <6 months. Other reasons more frequently identified by participants who weaned early were, "baby is always hungry," "maternal illness," "sucking and latching problems," "fatigue and stress," "nipple and/or breast pain," and "infant illness." Participants who weaned later than 6 months were more likely to cite "right time to wean" as their main reason for weaning.

**Table 3:** Primary reason for weaning according to infants' age at weaning

		Infants' age in months when no longer receiving any breastmilk				
		
Characteristic	Total (N = 1103)^a^ %	<1 (*n *= 469) %	1 to <3 (*n *= 322) %	3 to <6 (*n *= 132) %	6 to <9 (*n *= 97) %	9 to <12 (*n *= 83) %
Insufficient milk	34.5	36.7	31.1	37.1	35.1	30.1
Returning to Work†	31.4	12.6	58.7	48.5	23.7	13.3
Baby is always hungry†	14.1	21.5	11.8	4.6	4.1	7.2
Maternal illness†	11.7	17.3	7.8	5.3	6.2	12.1
Sucking/latching problems†	10.9	17.1	5.0	5.3	8.3	10.8
Fatigue/stress†	10.3	15.1	7.1	7.6	5.2	6.0
Inconvenient/too time consuming	8.9	10.9	7.1	9.1	8.3	4.8
Nipple/breast pain†	5.7	9.0	2.5	1.5	5.2	7.2
Infant illness†	4.8	9.4	2.5	0.8	0.0	0.0
Right time to wean†	3.5	0.2	0.0	6.1	19.6	13.3
Poor weight gain	1.9	3.0	1.6	0.0	1.0	1.2

† *p *< .001

^a ^16 participants did not have a stated reason for weaning

## Discussion

This study is the first study to report detailed breastfeeding practices among such a large cohort of Hong Kong breastfeeding mothers across the first year of the infant's life. Study findings are consistent with previous research in this population showing a short duration of breastfeeding and low rates of exclusive breastfeeding [17,22]. Just over 1/3 of babies in this study were receiving any breast milk beyond 3 months of age, and less than one-half of those were being exclusively breastfed. While breastfeeding initiation rates in Hong Kong have increased substantially over the past 10-15 years [15,16], less progress has been made on maintaining high rates of continued breastfeeding in the postpartum period. Results from this study highlight some possible explanations for this discrepancy and suggest some areas to address in further breastfeeding promotion.

There was a high prevalence of infant formula supplementation of breastfed babies across the first year of life among mothers in this study. In the multivariate analysis, the use of formula at 1 month of age was one of the strongest predictors of breastfeeding cessation before 3 months of age. Infant formula supplementation, in addition to early breast milk expression, are indicators of early breastfeeding problems [35]. Mothers who weaned early identified insufficient milk and sucking/latching problems as primary reasons, both of which substantially increase the rates of pumping and infant formula supplementation [36,37]. The high rates of infant formula supplementation and early milk expression, therefore, likely indicate that mothers are not receiving sufficient breastfeeding support in the immediate postpartum period. All obstetric wards in public hospitals in Hong Kong have board certified lactation consultants to assist breastfeeding mothers. The reality, however, is that because of heavy work demands in public hospitals, most lactation consultants work as staff nurses and often have little time to spend doing hands-on breastfeeding support with new mothers. Additionally, in Hong Kong, unlike other developed countries such as the United Kingdom, Australia, and Canada, routine home visits for new mothers in the immediate post-partum period are not provided. Comprehensive antenatal and postnatal care, as well as breastfeeding support, is available for free to Hong Kong mothers through the publicly funded Maternal and Child Health Centres, but that means that new mothers must themselves seek care and support. The early postpartum period is overwhelming for most new mothers and many have stopped breastfeeding before ever seeking support or help [38]. Breastfeeding education in the antenatal period alone is insufficient to prepare new mothers for the breastfeeding experience. Women in Hong Kong who attended a prenatal breastfeeding class reported that they were given much information on the benefits of breastfeeding but little on breastfeeding techniques and how to deal with problems when they arose [39]. Effective management of lactation problems in the early postpartum period is important to prevent early weaning [40]. Therefore, in order to promote the continuation of breastfeeding beyond the first few weeks postpartum, strong antenatal promotion of breastfeeding should be followed up with adequate support and hands-on assistance with breastfeeding in the postnatal period, both immediately after delivery and in the weeks that follow [40,41].

Returning to work was also a strong predictor of early weaning in this sample. In Hong Kong, over 75% of all women of childbearing age are employed full-time [28] with long working hours and a six-day work week still common. Similarly, 73.7% of mothers in this study reported that they were returning to work in the postpartum period. Government mandated maternity leave is granted for a maximum of 10 weeks, with at least two weeks having to be taken before the expected date of delivery [42]. Work has been frequently cited as a reason for early weaning from breastfeeding [17,41,43,44]. Breastfeeding mothers in Hong Kong have reported that breastfeeding after returning to work in not feasible for them, and thus with their long work days, they must wean before returning to work [23]. Although the Hong Kong government, primarily through the Department of Health, has been publicly and actively promoting breastfeeding over the past 10 years, few legislative actions have been taken. In contrast, in Mainland China, a less developed economy than Hong Kong, postpartum women are provided with a more supportive environment for breastfeeding due to government legislation. New mothers receive four months of paid maternity leave and receive two hours of breaks during their workday to breastfeed their infants [45]. Many new mothers in Hong Kong feel that the Hong Kong government, in addition to their rhetoric of breastfeeding promotion, must provide adequate breastfeeding support for new mothers and implement policies that assist mothers to continue breastfeeding [38].

Findings from this study also highlight the need for breastfeeding promotion and education to focus on the husband as new mother's breastfeeding behaviours are strongly influenced by the significant other's infant feeding preferences [46-48]. The majority of Hong Kong husbands, however, do not routinely attend antenatal check-ups or even antenatal education sessions with their wives [39]. Therefore, targeting breastfeeding education and promotion at husbands can be challenging. Studies in other populations however have shown that interventions targeting fathers demonstrate increases in the initiation of breastfeeding [49-52], the duration of breastfeeding [49,52], rates of exclusive breastfeeding [53], and perceived maternal support for breastfeeding [49,50].

While studies in other countries generally show that the duration of breastfeeding increases with both income and education [54,55], the pattern of breastfeeding among Hong Kong mothers differs somewhat. Mothers of both lower and higher education and income had longer breastfeeding duration than mothers of middle education and income levels. There are several possible explanations of this finding. First, low income and poorly educated mothers are more likely to be recent immigrants from Mainland China, where breastfeeding rates in most regions are higher than in Hong Kong [56]. Second, mothers of low income and education are less likely to be employed full-time and less likely to return to work in the immediate post-partum period and are thus able to breastfeed for longer. In the adjusted analysis, however, the effect of low income and low education on breastfeeding is mitigated by returning to work and other variables. Similar to many other countries, highly educated women with higher family incomes are more likely to have jobs with more flexible work environments, thus enabling these women to enjoy a longer maternity leave and/or continue breastfeeding after returning to work [57].

Overall, findings from this study highlight several key areas for breastfeeding promotion. In addition to focusing on increasing breastfeeding initiation, antenatal education should emphasize the importance of exclusive breastfeeding. It is also important to include the husband in breastfeeding promotion in the antenatal and early postpartum periods as spousal support is an important predictor of continued breastfeeding. Working class mothers who are returning to work should be provided with additional education and support in the early postpartum period. Many Hong Kong mothers who are returning to work believe that it is best to use some infant formula from the beginning so that the baby gets used to it and this will make the transition back to work will be easier [23]. Focus should be on how to continue breastfeeding after returning to work and if this is not possible, on the importance of maintaining exclusive breastfeeding for as long as possible. The risks of infant formula supplementation, both to the baby's health and to the process of breastfeeding, need to be reinforced to all mothers. Adequate postnatal support can address and correct many breastfeeding problems without the introduction of infant formula [58,59]. Without adequate resources and dedicated lactation consultants, however, this is difficult for already overburdened nursing staff in public hospitals and maternal and child health clinics.

### Strengths and limitations

To our knowledge, this study is one of the first to prospectively examine breastfeeding and weaning practices across the infant's first year for such a large cohort of Hong Kong Chinese women. As such, it provides a baseline for further research and can help to inform further breastfeeding promotion efforts. We prospectively recruited a large number of breastfeeding women from four geographically disparate hospitals across Hong Kong and followed them prospectively for one year or until weaned. We had a low drop-out rate and had breastfeeding follow-up data on 92.9% of our original sample.

This study, however, is not without some limitations. First, participants who were lost to follow-up after hospitalisation were more likely to have lower family income and a shorter duration of residence in Hong Kong. In this study, both of these variables were positively associated with continuation of breastfeeding. The most likely explanation as to why these women were lost to follow-up is that although they were Hong Kong residents, they live part of the time in Mainland China and hence returned to China to convalesce with their family after the birth. Second, our cohort participated voluntarily and we cannot rule out the possibility that participants with more positive breastfeeding attitudes and behaviours were more likely to participate. We do not have data available on those who chose not to participate in the study and what proportion of eligible mothers chose not to participate. Recent population based statistics on breastfeeding continuation in Hong Kong, however report similar rates of both any and exclusive breastfeeding [21], suggesting that the breastfeeding data collected in our sample is similar to the overall breastfeeding pattern in Hong Kong mothers. Third, although breastfeeding continuation data was collected prospectively, it was collected by maternal self-report in telephone follow-up interviews. It is possible, therefore, that mothers did not accurately report the duration of any and exclusive breastfeeding. Studies suggest, however, that maternal reporting of breastfeeding initiation and duration is accurate up to three years after the period of breastfeeding [60].

## Conclusions

The breastfeeding duration of Hong Kong mothers remains far short of WHO and DH recommendation of exclusive breastfeeding for 6 months and continued breastfeeding thereafter for up to 1 to 2 years. Few mothers exclusively breastfeed and the majority have stopped breastfeeding by 3 months postpartum. High rates of infant formula supplementation, inadequate early postpartum professional support to resolve breastfeeding problems, and a lack of family support for breastfeeding all contribute to early weaning in Hong Kong. Years of breastfeeding promotion programs have been successful in achieving high rates of breastfeeding initiation. The focus must now be on implementing adequate early postpartum support programs, both in the hospital and after the mother goes home, to enable mothers to continue breastfeeding for as long as possible.

## Competing interests

The authors declare that they have no competing interests.

## Authors' contributions

MT designed the study, obtained funding, performed data analysis and wrote the first draft of the paper. DF contributed to the study design, assisted with data analysis and critically reviewed and revised the final draft of the paper. KW assisted with data analysis and critically reviewed and revised the final draft of the paper. IL, EW, AS, CL, and JD contributed to the study design and critically reviewed and revised the final draft of the paper. The manuscript has been read and approved for publication by all authors.

## Pre-publication history

The pre-publication history for this paper can be accessed here:

http://www.biomedcentral.com/1471-2393/10/27/prepub
